# Urease in acetogenic *Lachnospiraceae* drives urea carbon salvage in SCFA pools

**DOI:** 10.1080/19490976.2025.2492376

**Published:** 2025-04-15

**Authors:** Isaac J. Firth, Marissa A.R. Sim, Bradley G. Fitzgerald, Ailish E. Moore, Christian R. Pittao, Connor Gianetto-Hill, Samantha Hess, Autumn R. Sweeney, Emma Allen-Vercoe, Matthew T. Sorbara

**Affiliations:** Department of Molecular and Cellular Biology, University of Guelph, Guelph, Canada

**Keywords:** Urease, urea, *Lachnospiraceae*, short-chain fatty acids, acetate, urea-derived acetate production, microbiota, acetogenesis, *Blautia*, acidification, butyrate, cross-feeding, *Anaerostipes*

## Abstract

The gut microbiota produces short-chain fatty acids (SCFA) and acidifies the proximal colon which inhibits enteric pathogens. However, for many microbiota constituents, how they themselves resist these stresses is unknown. The anaerobic *Lachnospiraceae* family, which includes the acetogenic genus *Blautia*, produce SCFA, are genomically diverse, and vary in their capacity to acidify culture media. Here, we investigated how *Lachnospiraceae* tolerate pH stress and found that subunits of urease were associated with acidification in a random forest model. Urease cleaves urea into ammonia and carbon dioxide, however the role of urease in the physiology of *Lachnospiraceae* is unknown. We demonstrate that urease-encoding *Blautia* show urea-dependent changes in SCFA production, acidification, growth, and, strikingly, urease encoding *Blautia* directly incorporate the carbon from urea into SCFAs. In contrast, ureolytic *Klebsiella pneumoniae* or *Proteus mirabilis* do not show the same urea-dependency or carbon salvage. In agreement, the combination of urease and acetogenesis functions is rare in gut taxa. We find that *Lachnospiraceae* urease and acetogenesis genes can be co-expressed in healthy individuals and colonization of mice with a ureolytic *Blautia* reduces urea availability in colon contents demonstrating *Blautia* urease activity *in* *vivo*. In human and mouse microbial communities, the acetogenic recycling of urea carbon into acetate by *Blautia* leads to the incorporation of urea carbon into butyrate indicating carbon salvage into broader metabolite pools. Altogether, this shows that urea plays a central role in the physiology of health-associated *Lachnospiraceae* which use urea in a distinct manner that is different from that of ureolytic pathogens.

## Introduction

The human gut microbiota is a diverse community of microorganisms that collectively benefit the host. In the proximal colon, fermentation of carbohydrates by resident anaerobes contributes to an environment that is slightly acidic and high in short-chain fatty acids (SCFA), with the most abundant being acetate.^1^ SCFA contribute to several beneficial functions of the microbiota.^2,3^ For example, concentrated SCFA at a slightly acidic pH can inhibit the replication of pathogenic bacteria as one mechanism of colonization resistance.^4^ In *Enterobacteriaceae*, including antibiotic resistant strains of *Klebsiella pneumoniae* and enteric pathogens like *Salmonella enterica* Typhimurium, the high SCFA/low pH combination triggers intracellular acidification at the environmental pH of the proximal colon.^3,5,6^ Antibiotic treatment that disrupts the microbiota increases environmental pH and decreases SCFA levels, indicating that this environment is established by the microbiota.^5^ However, the mechanisms by which the microbiota withstand these SCFA/pH stresses are not well understood.

Humans produce urea to process ammonia, a toxic product of nitrogen metabolism.^7,8^ While most urea is excreted in urine, approximately 20% is transported into the gut where it is cleaved by microbial urease.^7^ Urease is a multimeric enzyme that hydrolyzes urea into carbon dioxide and two ammonia molecules, raising the local pH.^9^ Mammals do not encode urease enzymes, so all urea hydrolysis in the gut is mediated by microbial urease. Urease is a well-characterized virulence factor;^9–12^ in some pathogens, including *Proteus mirabilis*, urease can function in a nitrogen acquisition role, while in others such as *Helicobacter pylori*, urease production is primarily an acid-stress response.^10,13,14^ Interestingly, while the overall abundance of urease genes is not altered between healthy individuals and individuals with Inflammatory Bowel Disease (IBD), there is a shift in the bacterial populations encoding urease with increases in urease-encoding *Enterobacteriaceae* compared to other commensals in IBD.^15^ It is currently unknown whether urease has the same function in gut commensals as in *Enterobacteriaceae*.

In addition to its role in pathogenesis, urease is also encoded by commensal bacteria in the gut.^8,16,17^ While the role of urease in pathogens and virulence is well established, its importance to the physiology of commensal gut microbes is a current focus of research. Indeed, investigation into urease-encoding isolates of *Bifidobacterium*^17^ and *Streptococcus*^18^ species have been performed which revealed changes in metabolism during urea supplementation. For these microbes, these shifts in metabolism are linked to nitrogen metabolism consistent with ammonia release and salvage.^17,18^ While multiple pathways for the assimilation of urea nitrogen are known,^19^ the fate of the urea carbon is less well characterized.

The *Lachnospiraceae* are a diverse family of anaerobes that produce SCFA and thus can contribute to colonization resistance.^20^ Some *Lachnospiraceae*, including members of the genus *Blautia*, are acetogens.^21^ Acetogenesis, encoded by the Wood-ljungdahl pathway (WLP), allows microbes to produce acetate from two carbon dioxide molecules.^22^ Interestingly, some *Blautia* lack formate dehydrogenase, an early WLP step, but can combine formate and CO_2_ to produce acetate.^21^ Overall, the WLP allows acetogens to produce high levels of acetate when growing heterotrophically.^22^ We previously observed that many *Lachnospiraceae* and *Blautia* strongly acidify culture media, but that acidification varies at a species and strain level, suggesting variation in pH stress responses and acid production.^20^ pH stress responses have been characterized across broad collections of isolates revealing varying pH tolerance across phylogenetically diverse microbes.^23^ However, the mechanisms by which *Lachnospiraceae*, and *Blautia* specifically, resist SCFA/pH stresses and the impacts of this stress on acetogenesis are not understood.

Given the importance of SCFA production and pH regulation in the gut, we investigated how *Lachnospiraceae*, including acetogenic *Blautia*, resist pH stress. We demonstrate that a subset of acetogenic *Blautia* encode a functional urease that promotes survival in acidic conditions and alters metabolism leading to increased SCFA production through direct incorporation of the carbon from urea into acetate, enabled by their acetogenesis pathway. Further, we found that the combination of acetogenesis and urease functions is rare across gut microbes, allowing *Blautia* to make specialized functional contributions to the gut environment. Indeed, drug-resistant isolates of the ureolytic pathogens *K. pneumoniae* and *P. mirabilis* did not show the same urea/urease dependency under conditions approximating the proximal colon. These results support a central role for urease in the physiology of health-associated *Lachnospiraceae* that differs from that in ureolytic pathogens. Finally, we demonstrate that the salvage of urea carbon into acetate by *Blautia* allows for the recycling of urea carbon into SCFA pools in both human and mouse fecal microbial communities.

## Methods

### Association of urease genes with acidification

To associate genes with acidification in *Lachnospiraceae*, we used a previously reported dataset of culture media acidification and whole-genome sequences of 273 isolates.^20^ Prokka was used to annotate the whole-genome sequences.^24^ The presence/absence of annotated protein coding sequences across the 273 isolates were used as predictors in a Random Forest model in regression mode to predict measured pH. In this approach, the 273 isolates were divided in training (70%) and test sets (30%). The caret R package (v. 6.0–94) was used to train model parameters with the following settings: resampling method: “repeatedcv”; 3 × 10-fold cross-validation in a grid search with mtry from 5:70. Subsequently, random forest models were built with the randomForest function, with ntree = 500, importance = T, and mtry determined by the bestTune in caret training. Model tuning and building were repeated with 10 independent test and training sets, and the average importance measures (IncMSE and IncNodePurity) were calculated for each protein product across the 10 replicates.

### *Bioinformatic analysis of urease in* Lachnospiraceae

**Figure 1.**
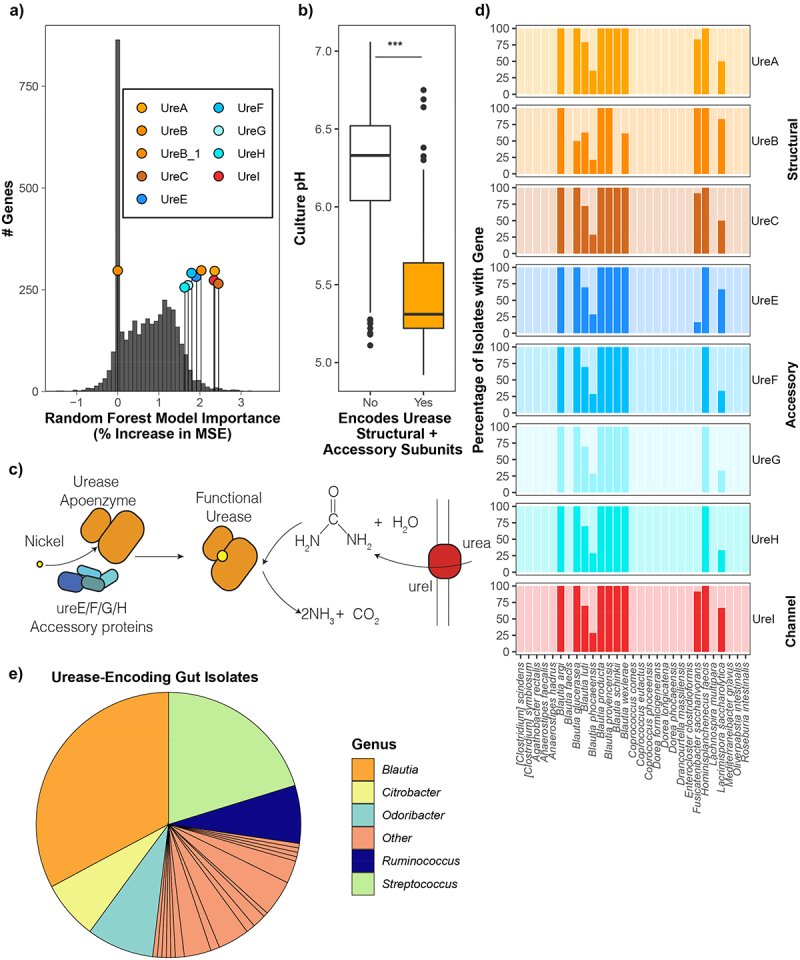
Urease is associated with increased acidification in *Lachnospiraceae*. a) %increase mean square error of annotated genes in a random forest model of acidification showing importance of urease genes. b) Culture acidification data from 273 *Lachnospiraceae* isolates stratified by urease gene presence. Two-tailed *t*test performed, *p* = 2.2e-16. c) Schematic of urease complex function. Structural subunits (orange) are assembled with the help of the accessory genes (blue), which also chaperone the Ni^2+^ ion into the active site of the enzyme. Urea can enter the cell through the urea channel UreI (red), where it is hydrolyzed by the urease enzyme. d) Percentage of isolates encoding urease genes across 273 *Lachnospiraceae* isolate biobank. e) Proportion of urease encoding isolates identified as *Blautia* and other genera in 4901 whole-genome sequenced gut isolates as described in methods.

*Urease and acetogenesis encoding and gene clusters*: Isolates in the *Lachnospiraceae* biobank^20^ with potential urease activity were determined by identifying isolates with at least two structural subunits and three required assembly factors, based on the minimum numbers of genes/subunits in reported urease gene clusters.^9^ To examine urease encoding in a broader collection of gut microbes, we annotated existing collections of whole-genome sequenced isolates^20,25–27^ using Prokka,^24^ and applied the same filter for structural and accessory urease genes. To determine the current taxonomy of isolates from these biobanks, the longest 16S rRNA gene from each assembled genome was used as the input in the isoTax function of isolateR^28^ with default settings. Isolates were aggregated at the family (Fig S1D) or genera level (Figure 1(e)). Urease gene clusters for representative isolates were constructed by selecting urease-encoding isolates of interest and plotting these clusters using the gggenes package in R.^29^ To analyze the co-occurrence of acetogenesis and urease genes in a global collection of cultured and non-cultured species, the UHGG collection was used.^30^ Urease presence was assessed as above, and acetogenesis was determined by the presence of both *acsC* and *acsD*.

*Structure and sequence analysis*: Structural predictions of urease enzymes and the urea channel protein from *Lachnospiraceae* and selected comparators were performed with the Alphafold 3 server.^31^ For UreI folding, Alphafold 3 server was used with the typical homohexamer stoichiometry for UreI channels. For urease folding, the Alphafold 3 server was used with enzyme stoichiometry of either a trimer of heterotrimers, or a trimer of heterodimers, based on whether that gene cluster encodes two or three structural subunits. To compare urease protein sequence homology, a concatenated amino acid sequence with all structural genes was generated. The approach of using concatenated amino acid sequences was taken to allow comparisons between species in which there is a single small subunit and species where the small subunit is divided into two coding sequences. The concatenated sequences were aligned using Clustal Omega and percent identities were calculated.^32^

*HMP transcriptomics*: Raw paired-end RNA-seq reads were obtained from the HMP portal client AWS servers and processed with Trimmomatic (v0.36) using default settings.^33^ Index files for the filtered sequences were generated using the bowtie2-build command to create a searchable reference index. Gene sequences for the large subunit of urease, acsC and acsD from across the 4901 gut isolates (described above) were used as input query sequences. Indexed reads were then mapped to query gene sequences using Bowtie2 (v2.4.4).^34^ Bowtie2 output was converted to BAM format using SAMtools (v1.13).^35^ Read counts were normalized to read depth and gene length to generate RPKM values, which were then aggregated at the genus level within each sample.

### Bacterial strains and growth

*Strains: Lachnospiraceae* isolates *Anaerostipes hadrus* MSK.9.9 (*Anaerostipes*), *Blautia* MSK.15.19 (*Blautia* A), *Blautia* MSK.23.78 (*Blautia* B) and *Blautia* MSK.20.66 (*Blautia* C), were isolated from human donors as described in.^20^ *Klebsiella pneumoniae* (Strain MH258),^36^ and *Proteus mirabilis* (Strain MH42F)^5^ are both drug resistant clinical isolates.

*Growth*: All *Lachnospiraceae* were streaked onto reduced brain-heart infusion (BD) agar plates supplemented with yeast extract (Thermo Scientific) and 0.1% L-cysteine (Thermo Scientific) (BHIS media) in an anaerobic chamber (Coy Laboratory Products), supplied with gas mix of 90% N_2_, 5% CO_2_ and 5% H_2_. Anaerobic chambers were maintained at atmospheric pressure and equipped with a COY dehumidifier system at factory settings (Coy Laboratory Products) to control moisture. Isolated colonies were picked and inoculated into 5 mL of reduced BHIS broth and grown overnight at 37°C. Overnight cultures were then used to inoculate the assay broths at 1:100 dilution (50 µL culture into 5 mL test broths). Viability of cultures was assessed using plate counts on BHIS agar and culture pH was recorded with a pH probe.

*Assay broths*: The various assay conditions were prepared as follows: i) pH: BHIS broth was either used at starting pH (pH 7) or pH was adjusted to 5.5 with hydrochloric acid. ii) Urea supplementation: pH adjusted broths were aliquoted and a freshly prepared 10 M urea solution (Bioshop Canada Inc.) was used to supplement broths with 2.5, 5.0, 7.5, 10, 20 mM urea. Addition of urea did not alter measured pH. iii) Urease inhibitor experiments: for conditions with flurofamide (Tocris biosciences),^37^ assay broths were supplemented with 3 µg/mL flurofamide dissolved in 80% DMSO, 80% DMSO as vehicle control, or no additional treatment. iv) ^13^C-labeled urea and formate: ^13^C-labeled urea (Sigma-Aldrich), urea (Bioshop Canada Inc.), ^13^C-labeled formate (Sigma-Aldrich) or formate (Fisher Scientific) were used to supplement BHIS broth at the indicated pH at a concentration of 10 mM. After preparation, all assay broths were then transferred to the anaerobic chamber to reduce overnight. For urease-complemented experiments, at the time of inoculation, 5 µL of Jackbean urease glycerol solution (Sigma-Aldrich) or *P. mirabilis* overnight culture was added to 5 mL of culture broth.

### Metabolomics

*Chemical ionization*: Targeted metabolomics was performed using methods previously reported in.^38^ Briefly, culture samples were collected and centrifuged at 13,000 *g*. Supernatants were collected in 200 µL aliquots and stored at −80°C until processing. Thawed samples were diluted with four volumes of ice-cold methanol (Fisher) containing internal standards of D3-acetate, D7-butyrate, and D6-succinate (Sigma-Aldrich). After mixing, samples were centrifuged at 13,000 *g* at 4°C for 18 min. 100 µL of supernatant was then transferred to 2 mL glass mass-spec vials, along with 100 µL borate buffer (pH 10, Sigma-Aldrich), 400 µL acetonitrile (Sigma-Aldrich) with 150 mM pentafluorobenzyl bromide (Sigma-Aldrich), and 400 µL hexane (Sigma-Aldrich). Samples were then incubated at 65°C for 1 h with shaking at 1500 rpm. After mixing, the hexane layer was transferred to new 2 mL glass mass-spec vials. Samples were analyzed using a GC-MS (Agilent 8890; Agilent 5977B) in negative chemical ionization mode with methane as the reagent gas and helium as the carrier gas. Mass Hunter Quantitative analysis (Agilent Technologies, v10.2.733.8) was used to measure peak areas for defined compounds. Peak areas were then normalized to internal standards, and a concentration was calculated for SCFA using a standard curve of known concentrations.

Abundance of labeled metabolites was calculated by normalizing the peak areas of each detected isotope to internal standards. Normalized values were then used to calculate the relative abundance of each isotope by dividing by the sum of all detected isotopes e.g. [M + 1]/([M]+[M + 1]+[M + 2]).

*Electron ionization*: Mouse colon contents collected after sacrifice were suspended to 100 mg/mL in 80% methanol supplemented with 1 mM DL-proline (Cambridge Isotope laboratories; DLM-2657-PK), 1 mM D_3_-lactate (Cambridge Isotope laboratories; DLM-9071-0.25), and 5 mM D_6_-succinate (Sigma; 488356), homogenized by vortexing, and stored at −80°C overnight. Samples were then centrifuged at 20, 000 × g for 10 min at 0°C. Supernatant aliquots of 100 μL were dried under nitrogen stream at 30°C for 2.5 h. 50 μL of 20 mg/mL methoxyamine (Sigma; 226904) dissolved in pyridine (Sigma; 270970) was added to dried samples followed by incubation in a thermomixer for 90 min at 1400 rpm, 30°C. Samples were allowed to cool to room temperature before 80 μL of derivatizing reagent (BSTFA +1% TMCS, Sigma; B-023) and 70 μL ethyl acetate (Sigma; 439169) were added to the samples, followed by incubation in the thermomixer for 1 h at 70°C, 1500 rpm. Samples were cooled to room temperature and then 400 μL ethyl acetate was added. 200 μL were transferred into GC-MS vials with inserts. Samples were analyzed using a GC-MS (Agilent 8890; Agilent 5977B) in electron ionization mode with helium as the carrier gas. Mass Hunter Quantitative analysis (Agilent Technologies, v10.2.733.8) was used to measure peak areas for defined compounds. Urea peak area was normalized to the internal standard of D_6_-succinate.

### Human fecal samples

*Stool sample collection*: Participants were recruited from Guelph, Canada through posters placed around the University of Guelph main campus. The collection of these stool samples was approved by the Research Ethics Board at the University of Guelph (REB#22-08-017). Respondents to the advertisements were only supplied a collection kit if they signed the consent form and self-reported they met the following requirements at the time of donation: aged between 18 and 28, no history of antibiotic usage within 1 year prior to recruitment, nonsmoking, no chronic diseases, including obesity, autoimmune diseases, type 1 or type 2 diabetes, or any gastrointestinal diseases experienced personally or by an immediate family member, no history of symptomatic viral or bacterial infection within 6 months, and not taking any doctor-prescribed medications. A single stool sample was collected at only one time point from each participant. Following donation, the samples were stored on wet ice during transport to the lab (less than 1 h) where the samples were transferred to −20°C. The stool samples were thawed anaerobically and split into 5 g aliquots to limit freeze thaw cycles before refreezing at −20°C for long-term storage.

*Metagenomic Sequencing*: DNA extraction was performed using 0.2 g of stool with the QIAmp Power Fecal Pro DNA Kit (Qiagen, USA). Manufacturer’s directions were followed with the following modifications: After samples were vortexed for 20 min using the PowerBead Pro Tubes, the samples were incubated in a 95°C water bath for 10 min, sonicated for 5 min, followed by the addition of Proteinase K (Thermo Fisher) to a final concentration of 19.5 mg/mL and incubation at 70°C for 10 min. The protocol then proceeded according to the manufacturer’s directions. Quantification of the extracted DNA was measured using Qubit Fluorometer 2.0 (Invitrogen, USA). Extracted DNA samples were sent to CosmosID who performed Illumina library preparation for shallow shotgun metagenomic sequencing through paired-end Illumina sequencing. Sequencing was performed to a depth of around 3 million reads per sample.

*Processing Metagenomic Sequencing Data*: The sequencing files were first processed using KneadData version 0.10.0 using the recommended settings. Sequences matching to the hg37 and human contamination reference database (version 0.1) were removed at this step to limit the contamination of human DNA in future processing steps. Taxonomic classification was performed using MetaPhlAn3 (version 3.0.14) with recommended settings.^39^ Functional profiling was performed using HUMAnN3 (version 3.9) using the ChocoPhlAn and UniRef90 databases for annotation.^39^ The output gene abundance table from HUMAnN3 was then regrouped based on KEGG Orthogroups.

*Ex vivo Assay*: Frozen fecal samples were thawed at room temperature under anaerobic conditions before being suspended at 100 mg/mL in pre-reduced PBS. After being homogenized by vortexing, suspensions were left to settle for approximately 20 min. *Blautia* isolates were grown on BHI agar plates to a dense lawn, and plate-scrapings were resuspended in reduced PBS at an OD600 of 1. Fecal supernatant samples were withdrawn in 1 mL aliquots, and 500 μL of *Blautia* PBS suspension was added where applicable. Suspensions were then spun for 5 min at 5000 *g*, followed by resuspension in 1 mL of growth media, and incubation at 37°C. Samples were taken at 6 h after resuspension in BHI, and subjected to GC-MS analysis.

### Mouse experiments

*Housing and gavage*: All animal procedures were approved by the University of Guelph Animal Care Committee and were performed at the Central Animal Facility (AUP #5226). 6–8-week-old C57BL/6 female mice were purchased from Jackson Laboratories and allowed to acclimatize in the facility for 7 days before use. Mice were separated into treatment groups (2–4 mice/cage). Select groups received ampicillin-treated autoclaved water at a concentration of 0.5 g/L for 4 days with fresh ampicillin-treated autoclaved water being added at day 2. After 4 days of antibiotics, mice transferred to cages with antibiotic-free autoclaved water for the remainder of the experimental timeline. Two days post-antibiotic treatment, mice were administered 100 μL of PBS or bacterial suspension (10^7^ to 10^8^ CFU) *Blautia A (MSK.15.19)* or *Blautia A (MSK.15.19)* + *Anaerostipes* by oral gavage. Administration of bacteria or PBS was performed for three consecutive days. Mice were euthanized 2 h after the final round of gavage and colon contents were harvested. Fecal pellets were collected pre- and post-antibiotic treatment and during colonization as shown in Figure 7(c). Freshly collected fecal pellets were transferred to an anaerobic chamber and suspended at 100 mg/mL directly in reduced BHI with 10 mM ^13^C-urea. Resuspended cultures were then incubated anaerobically at 37°C for 3 h before collection and GC-MS analysis.

### Statistics

Statistics were performed using GraphPad Prism 10. Viability data (CFU/mL values) were log10 transformed before statistical calculations. All data were assessed for normality and equality of variance using Shapiro-Wilk and Brown-Forsythe tests, respectively. No outliers were excluded from statistical calculations. Urease-stratified culture pH data was analyzed using two-tailed *t*-test. Ordinary one-way ANOVA calculations were performed with Dunnett’s multiple comparisons correction as post-hoc. For experiments with multiple concentrations of urea, means of each concentration were compared to that of baseline media. For experiments with urease inhibitor, DMSO and fluorofamide groups were compared to the mean of their respective control values (baseline or urea supplemented). For experiments with labeled urea, relative abundance values of urea and ^13^C-labeled urea conditions were compared to the baseline condition. For experiments where only one concentration of urea was tested, two-tailed *t*tests were performed. For relative abundance data for mouse ex vivo assays, one-sided *t*-tests were performed against the calculated or observed natural abundance of the isotope. For concentrations of mouse *ex vivo* assays, ordinary one-way ANOVA was performed with Dunnett’s multiple comparisons correction as post-hoc, comparing gavage groups to vehicle control (PBS). All calculated values and raw data are presented in Table S1.

## Results

### Lachnospiraceae *urease is associated with acidification*

The combination of concentrated SCFA and acidification of the proximal colon contributes to colonization resistance and helps shape the composition of the microbiota.^4,5^ We previously found that *Lachnospiraceae* variably acidify culture media,^20^ but the genomic and functional differences underpinning this variation were not investigated. Given the importance of pH regulation in the gut, we sought to identify associated genomic features using this acidification dataset.^20^ We employed a Random Forest (RF) model based on gene presence/absence, which explained an average 83% of the variance in acidification across independent test/training set pairs (Fig S1ABC). We then examined which genes were most important in the RF models. Interestingly, we observed that multiple subunits of urease are important RF features (Figure 1(a)). In agreement, stratifying *Lachnospiraceae* culture acidification data by the presence of structural urease subunits demonstrates that urease-encoding *Lachnospiraceae* reached significantly lower pH compared to urease-negative *Lachnospiraceae* (Figure 1(b)).

Production of urease requires urease structural subunits, and the UreE, UreF, UreG, and UreD/UreH accessory proteins that assemble the functional enzyme and chaperone the nickel ion into the active center^40^ (Figure 1(c)). Therefore, we examined the distribution of these genes across *Lachnospiraceae* isolates (Figure 1(d)). This revealed that many, but not all, members of the *Blautia* genus in this collection encode the required components for urease (Figure 1(d)). Interestingly, several *Blautia* species show strain-level heterogeneity in urease gene presence (Figure 1(d)). Next, we asked if urease encoding *Blautia* were frequent in a broader collection of gut isolates. This revealed that, while most *Lachnospiraceae* do not encode urease (Fig S1D), *Blautia* were the most frequent urease-encoding genera (Figure 1(e)).

**Figure 2.**
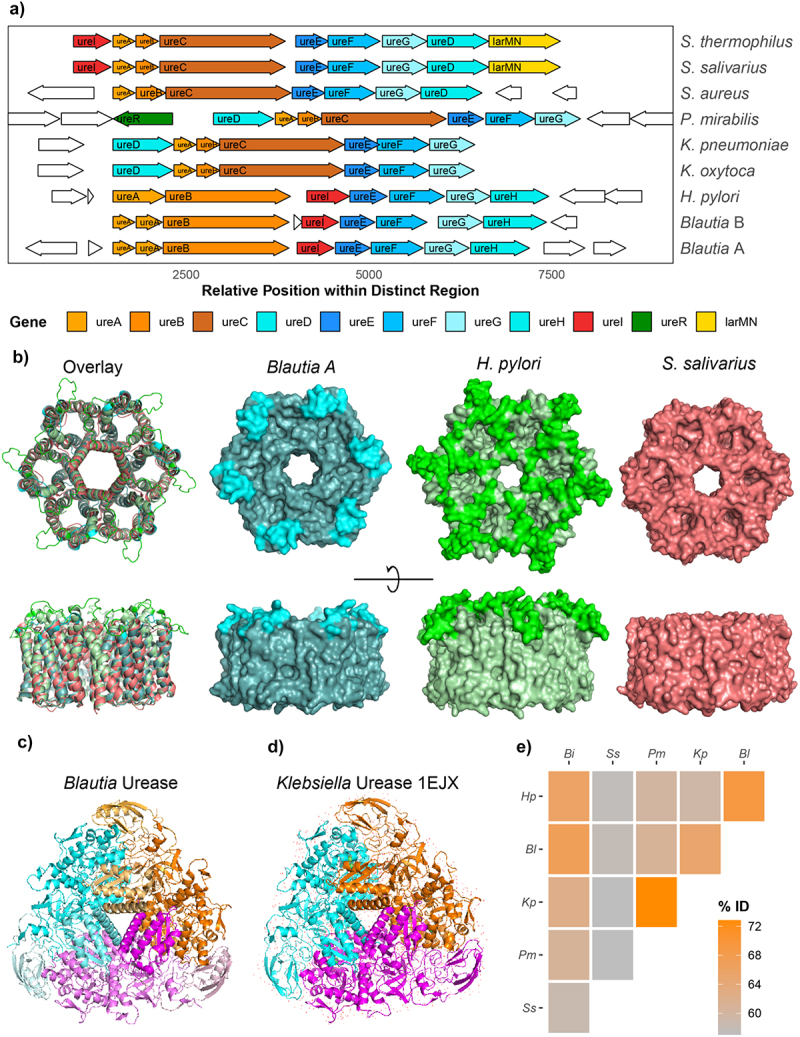
*Blautia* encode a urease gene cluster with similarity to *Helicobacter*. a) Urease gene clusters of *Blautia* isolates and other ureolytic bacteria. b) Alphafold predicted UreI structures of *Blautia* (blue), *H. pylori* (green), and *S. salivarius* (red). Highlighted regions show the loop domains in HpUreI and BlUreI that are absent in SsUreI. c) Alphafold predicted structure of the urease enzyme of *Blautia*. The urease enzyme is a trimer of oligomers, with each oligomer shown in a different color. d) Urease structure of *Klebsiella aerogenes* captured by X-ray diffraction (PDB ID: 1EJX). e) Amino acid sequence homology of concatenated urease structural proteins.

In ureolytic pathogens, genes encoding structural subunits and accessory proteins are arranged in a single gene cluster that can vary between species.^9^ In urease-encoding *Blautia*, isolates harbor a urease gene cluster with a conserved structure (Figure 2(a)). Comparing the *Blautia* urease gene cluster with that of ureolytic *Klebsiella pneumoniae*, *Proteus mirabilis*, *Helicobacter pylori*, *Streptococcus thermophilus*, and *Streptococcus salivarius* demonstrated that the *Blautia* urease cluster is similar to the *H. pylori* cluster in gene arrangement (Figure 2(a)). For example, unlike *P. mirabilis, Blautia* do not encode the UreR regulatory protein (Figure 2(a)). In *Blautia*, genes for nickel transport are located elsewhere in the genome, in contrast to *Streptococcus* and *Bifidobacterium longum* subsp. *infantis* that encode a nickel transporter adjacent to the accessory urease genes.^16,41^ While the overall synteny of the *Blautia* gene cluster is similar to *H. pylori*, only *H. pylori* encodes ureA as a single protein (Figure 2(a)).

*Blautia, H. pylori* and *S. salivarius* all encode the urea channel, ureI (Figure 2(a)). In *H. pylori* and *S. salivarius*, this channel allows for rapid uptake of urea during acid stress, in line with a primary role for urease as an acid stress or acclimation response.^42^ We examined the predicted structure of *Blautia* ureI (BlUreI) (Figure 2(b)). BlUreI was highly similar to predicted structures of both the *H. pylori* UreI (HpUreI) and *S. salivarius* ureI (SsUreI) channels and existing cryo-EM structure of HpUreI (Figure 2(b) ; *not shown*).^43^ HpUreI is pH gated by extracellular loops which are absent in non-gated SsUreI,^42^ and, in agreement, in the predicted structures, extracellular loops are present for HpUreI but not SsUreI (Figure 2(b)). Interestingly, BlUreI is predicted to have a distinct loop structure on the extracellular face (Figure 2(b)).

Next, we examined the structural components of urease. Predicted structures of *Blautia* urease subunits were consistent with known structures of *K. pneumoniae* and *H. pylori* urease enzymes (Figure 2(c,d)), and *data not shown*). Unexpectedly, sequence comparisons of the structural subunits of urease revealed that the *H. pylori* subunits are more similar to *Blautia* subunits than other members of the Pseudomonadota that are more closely related taxonomically (Figure 2(e)).

Altogether, this analysis indicates that some *Blautia* encode a urease gene cluster that shows structural and sequence similarity to urease in *H. pylori*. Based on the pH-driven role of urease in *H. pylori*, we hypothesized that urease in *Blautia* might function in a similar manner.

### *Urea regulates growth of urease-positive* Blautia *under acidic conditions*

**Figure 3.**
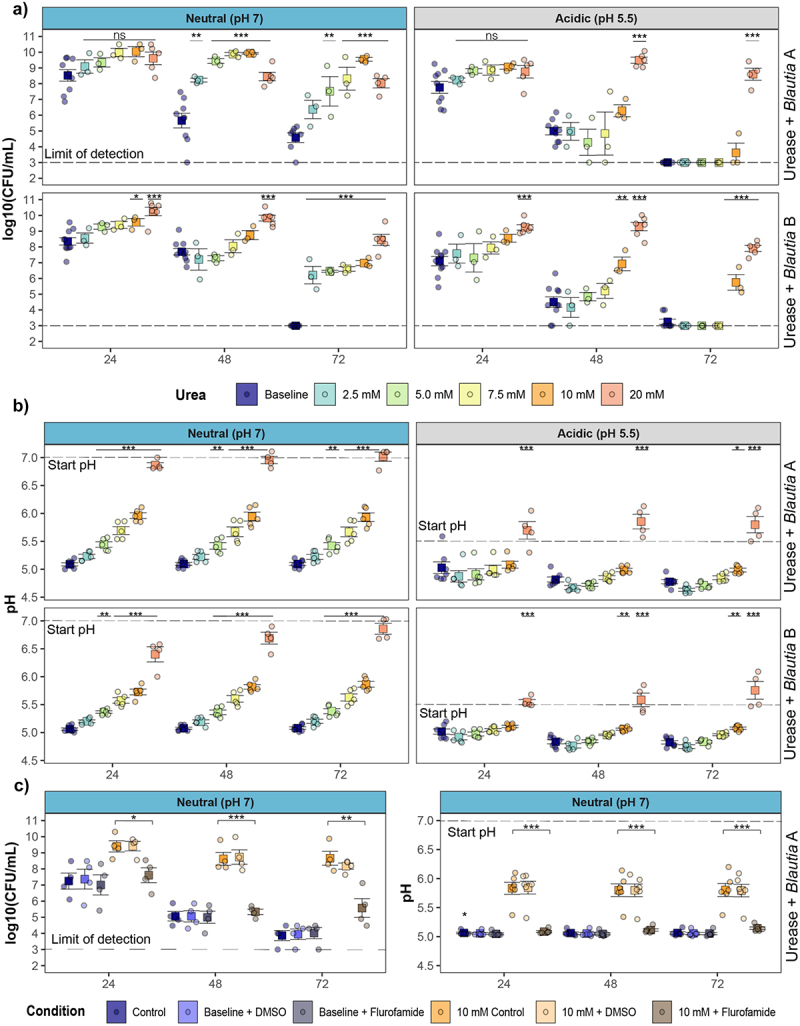
Urease regulates viability and pH modulation in urease-positive *Blautia* isolates. a) viability and b) acidification of urease-encoding *Blautia* isolates in neutral or acidic starting pH conditions with indicated urea supplementation. c) Viability and acidification of urease positive *blautia* in neutral conditions with urea supplementation and urease inhibitor (flurofamide) or vehicle (DMSO). Data points represent individual samples from independent replicates. (*p < 0.05, **p < 0.01, ***p < 0.001).

We tested the hypothesis that urease alters *Blautia*’s survival and growth in acidic conditions approximating the pH of the proximal colon. The effect of urea supplementation on the growth and viability of two urease-positive and one urease-negative *Blautia* was measured at either neutral or acidic conditions. In neutral media, without urea supplementation, *Blautia* grow rapidly and acidify the culture media, which is followed by decreased viability (Figure 3(a,b) – Left Panel). Similarly, in acidic media, without urea supplementation, *Blautia* viability significantly decreases at later timepoints (Figure 3(a)). Strikingly, urea supplementation increased viability of urease-positive *Blautia*, in a concentration-dependent manner, for both pH conditions (Figure 3(a)). Neutral conditions showed improved survival with as little as 2.5 mM urea supplementation, while acidic-start cultures did not show significant differences until 10 mM urea was added. In contrast, urea supplementation did not alter the growth of urease-negative *Blautia* under either neutral or acidic starting conditions (Fig S2A – Top Panel).

Next, we examined if urea supplementation altered pH regulation. Under neutral starting conditions, urease-positive *Blautia* isolates acidified culture media to ~pH 5, and urea supplementation resulted in a dose-dependent pH increase (Figure 3(b) – Left Panel). Conversely, under acidic conditions, urease-positive isolates acidified media to pH 5, but pH was not significantly altered with urea supplementation for all but the highest concentration of urea (Figure 3(b) – Right Panel). As expected, urea supplementation did not alter pH under any conditions for urease-negative *Blautia* (Fig S2A – Bottom Panel).

Next, we asked if the urea-dependent changes in viability and acidification were dependent on urease activity. Treatment with flurofamide, a potent urease inhibitor,^37^ but not the DMSO control, reversed the urea-dependent changes in viability and acidification in urease-positive isolates (Figure 3(c), S3BC).

Together, this demonstrates that a subset of *Blautia* encode a functional urease that increases viability and mediates pH regulation in the presence of physiological concentrations of urea.

### Effects of urea on growth and pH in ureolytic Klebsiella and Proteus

**Figure 4.**
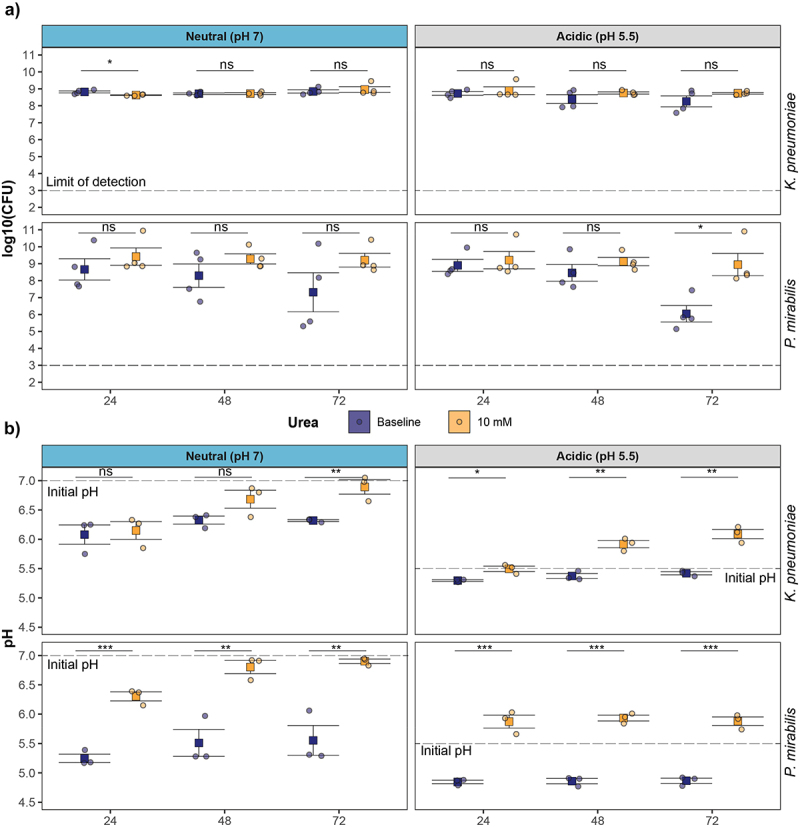
Regulation of viability and acidification in urease-positive *K. pneumoniae* and *P. mirabilis*. a) Viability and b) Acidification of ureolytic pathogens in neutral or acidic starting pH conditions with 10 mM urea supplementation. Data points represent individual samples from independent replicates. (*p < 0.05, **p < 0.01, ***p < 0.001).

Next, we compared the effects of urea supplementation on *Blautia* with two multi-drug resistant ureolytic Pseudomonadota: a carbapenem-resistant *K. pneumoniae* (MH258)^36^ and *P. mirabilis* (MH42F).^5^ In contrast to *Blautia*, *Klebsiella* MH258 viability was maintained over 72 h in neutral and acidic starting conditions, and urea supplementation did not increase viability (Figure 4(a)). *Proteus* MH42F viability was maintained over the first 48 h but declined at 72 h independent of starting culture pH, although less dramatically than for *Blautia* (Figure 4(a)). This decrease was alleviated with urea supplementation (Figure 4(a)). Overall, the effect of urea supplementation on pH differed between urease-encoding *Blautia* (Figure 3(b)) and the Pseudomonadota strains (Figure 4(b)). For *Klebsiella* MH258, in neutral starting media, urea supplementation did not increase the pH within 24 h, but did increase pH under acidic starting conditions (Figure 4(b)). For *Proteus* MH42F, in both initial media conditions, urea supplementation increased pH (Figure 4(b)). Despite limited effects on viability (Figure 4(a)), urea supplementation did significantly increase the pH in *Klebsiella* and *Proteus* cultures to level of (neutral) or above (acidic) the initial pH conditions. Overall, this indicates that urease increases *Blautia* viability in conditions where *Klebsiella* and *Proteus* do not depend on urease for survival and reveals differences in urease-dependent pH modulation between the studied strains of these species.

### *Urea increases SCFA production under acidic conditions by urease-positive* Blautia

**Figure 5.**
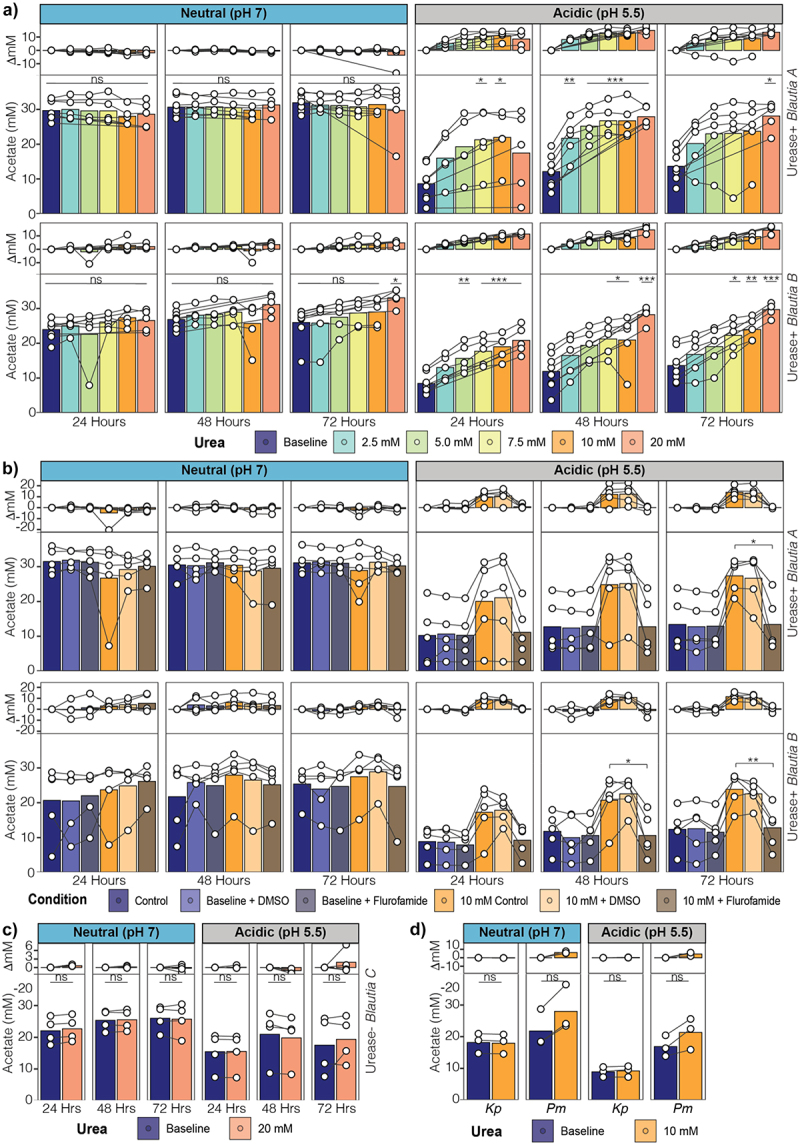
*Blautia* increase acetate production in response to urea. a) Production of acetate by urease encoding *Blautia* in neutral or acidic starting pH with various urea supplementations and b) Urease inhibitor. c) Production of acetate by urease-negative *Blautia* C supplemented with urea. d) Production of acetate by ureolytic pathogens supplemented with 20 mM urea. Data points represent individual values, lines connect samples within experimental replicates. The change in concentration (upper portion of graph) was calculated within each experimental replicate, between the treatment conditions. (*p < 0.05, **p < 0.01, ***p < 0.001).

*Blautia* encode the Wood-Ljungdahl pathway (WLP) of acetogenesis, which increases acetate production.^21^ Therefore, we asked if urea supplementation altered SCFA production. Without urea supplementation, ureolytic *Blautia* produced increased acetate and succinate in neutral compared to acidic conditions (Figure 5(a), S3A). Under neutral conditions, urea supplementation did change acetate or succinate production despite increased viability at most doses (Figure 5(a), S3A). In contrast, in acidic conditions, urea supplementation increased the release of acetate and succinate in a dose-dependent manner. Interestingly, urea supplementation at concentrations (2.5, 5.0, 7.5 mM) that did not significantly increase viability, still increased SCFA production under acidic conditions (Figure 5(a), S3A). Inhibition of urease with flurofamide reversed the increases in SCFA production by urease-positive *Blautia* with urea supplementation (Figure 5(b), S3B). Urease-negative *Blautia*, which do not produce succinate, did not increase acetate production with urea supplementation under either pH condition (Figure 5(c)). In contrast to ureolytic *Blautia*, ureolytic *Klebsiella* MH258 and *Proteus* MH42F did not show the same urea- or pH-specific enhanced production of acetate or succinate (Figure 5(d), S3C). Under these conditions, the tested *Blautia* strains did not produce butyrate or propionate (Figure S3D).

This demonstrates that urea supplementation increases *Blautia* SCFA production and that changes in SCFA production cannot be fully accounted for by changes in viability.

### Acetate production from urea and formate-derived carbon

**Figure 6.**
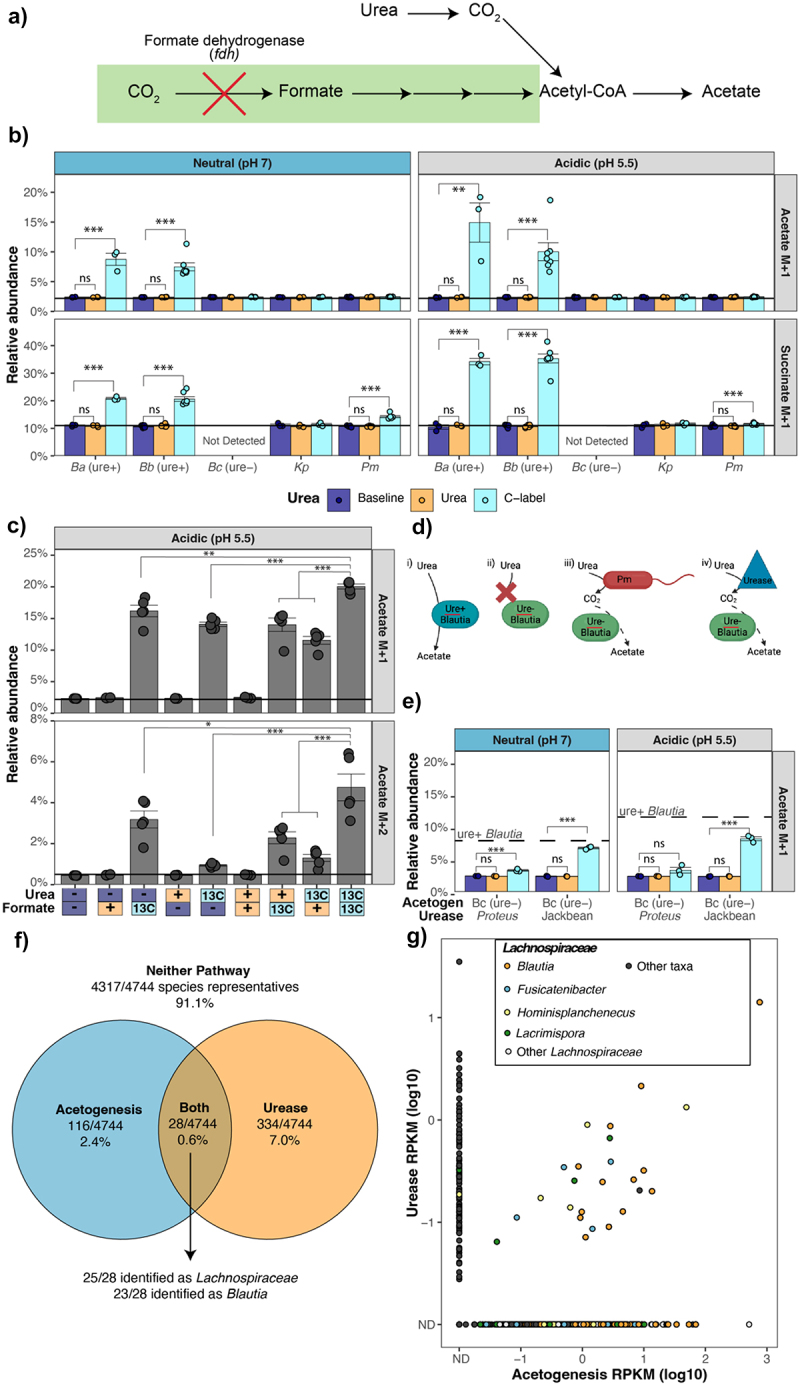
*Blautia* incorporate urea carbon into SCFA. a) Schematic of the Wood-Ljungdahl pathway of acetogenesis showing incorporation of urea-derived CO_2._ schematic shows an absence of formate dehydrogenase which has been previously reported in *Blautia* and describes *Blautia* A and B. b) Relative abundance of heavy (+1) isotopes of acetate and succinate when supplemented with ^13^C-labeled urea. c) Relative abundance of ^13^C-labeled acetate from urease-positive *Blautia* a cultures supplemented with ^13^C-labeled urea, ^13^C-labeled formate, or both. Solid horizontal lines indicate the natural or baseline abundance of the given isotope. d) Schematic showing: i) Urease-encoding *Blautia* incorporate urea-derived carbon into acetate. ii) Urease-negative *Blautia* do not respond to urea. iii, iv)Putative pathways showing urease-negative *Blautia* incorporating urea carbon into acetate following release through urease activity in *P. mirabilis* (iii) or exogenous urease (iv). e) Relative abundance of heavy (+1) isotopes of acetate from cultures of urease-negative *Blautia* C with urease activity provided by purified Jackbean urease or *P. mirabilis* in coculture. Dashed horizontal lines indicate the average relative abundance of acetate urease-positive *Blautia* cultures with C-label urea calculated from values in B. Data points represent individual samples from independent replicates. (*p < 0.05, **p < 0.01, ***p < 0.001). f) Presence of acetogenesis pathway (*acsC* and *acsD*) and urease (structural subunits and accessory proteins) across species representatives in the UHGG collection. g) Transcriptomic reads from HMP healthy donors aligned with Bowtie2 to urease and acetogenesis genes from 4901 gut isolates as described in methods. All genera labelled are members of *Lachnospiraceae*. (*p < 0.05, **p < 0.01, ***p < 0.001).

Given our observation that acetogenic *Blautia* isolates release more acetate with urea supplementation, in some cases independent of altered viability, we asked if the carbon in urea was directly incorporated into the acetate being released through acetogenesis (Figure 6(a)). In agreement, supplementation of cultures with ^13^C-labeled urea revealed that urease-positive *Blautia* isolates incorporate the urea carbon into acetate and succinate (Figure 6(b)). Interestingly, under acidic conditions, a greater fraction of released acetate and succinate was derived from the carbon in urea than in neutral conditions (Figure 6(b)). As expected, urease-negative *Blautia* did not incorporate the carbon from urea into acetate (Figure 6(b)). Similarly, ureolytic but non-acetogenic *Klebsiella* and *Proteus* strains did not incorporate carbon from urea into acetate (Figure 6(b)). There was a low level of succinate labeling in *P. mirabilis* MH42F cultures, but this occurred at neutral conditions rather than acidic, in contrast to the pattern observed with *Blautia* isolates (Figure 6(b)).

Some *Blautia*, including the strains tested here, lack formate dehydrogenase, the first step of the methyl branch of the WLP.^21^ In this case, acetate can be produced with formate as a substrate (Figure 6(a)). Therefore, we asked if formate could be combined with the carbon from urea to produce acetate. Consistent with this, supplementation with ^13^C-labeled formate increased ^13^C-labeled acetate production (Figure 6(c)). As expected, addition of unlabeled urea did not further increase formate-derived ^13^C-labeling of acetate. Similarly, addition of unlabeled formate did not increase ^13^C-labeling of acetate derived from ^13^C-labeled urea (Figure 6(c)). However, when both ^13^C-labeled formate and ^13^C-labeled urea are provided, the levels of ^13^C-labeled +1 and + 2 acetate significantly increase (Figure 6(c)). The increase in +2 acetate is greater than the sum of the +2 acetate levels present in ^13^C-labeled urea or ^13^C-labeled formate individually (Figure 6(c)). This is consistent with *Blautia* combining the urea carbon with formate to produce acetate using the acetogenesis pathway.

Next, we asked if co-expression of urease and acetogenesis by the same microbe was necessary to salvage carbon from urea into acetate (Figure 6(d)). First, we tested if urease activity provided by ureolytic *P. mirabilis* would enable incorporation of the urea carbon into acetate by a urease-negative, acetogenic *Blautia* isolate (Figure 6(d)). Under these conditions, incorporation of urea carbon was detectable but much lower than with ureolytic *Blautia* (Figure 6(e)). Direct supplementation of an excess level of jackbean urease into the cultures did support incorporation of urea carbon by urease-negative *Blautia* (Figure 6(e)). This indicates that having urease and acetogenesis functions in different microbes results in less efficient incorporation of urea carbon into acetate, but that urease-negative *Blautia* are still capable of incorporating urea carbon if it is released at high levels in the extracellular environment (Figure 6(d,e)).

To determine the frequency of acetogenesis and urease pathway co-occurrence, we used the large Unified Human Gastrointestinal Genome (UHGG) collection.^30^ Across the UHGG, 91% of species lack either urease or acetogenesis genes, while 7.1% encode urease and 2.5% encode core genes for acetogenesis (Figure 6(f)). Interestingly, only 0.6% of species (28 species) groups encoded both urease and acetogenesis pathways. Strikingly, the majority of these were members of *Lachnospiraceae*, and 23/28 were assigned to *Blautia* (Figure 6(f)). Altogether, this shows that, outside of a subset of *Blautia*, co-occurrence of urease and acetogenesis pathways is rare.

Next, we examined the expression of urease and acetogenesis genes *in* *vivo* using metatranscriptomic datasets from healthy individuals.^44^ Reads were mapped to urease and acetogenesis genes from diverse microbes in isolate biobank collections^20,25–27^ (Figure 6(g)), and consistent results were obtained with the UHGG collection (*data not shown*). Overall, *in* *vivo* expression of urease and acetogenesis genes was common with 97% of communities expressing detectable levels of acetogenesis genes and 55% expressing urease. Furthermore, this analysis revealed that, in a subset of healthy donors, *Lachnospiraceae* and *Blautia* urease and acetogenesis genes are co-expressed (Figure 6(g)). Consistent with our findings that *Lachnospiraceae* and *Blautia* are the predominant microbes encoding both pathways (Figure 6(f)), other members of *Lachnospiraceae* and *Blautia* were the primary taxa that mapped both urease and acetogenesis reads (Figure 6(g)). Altogether, this shows that expression of these pathways occurs in human microbiomes.

### Recycling of urea carbon in SCFA pools by microbial communities

**Figure 7.**
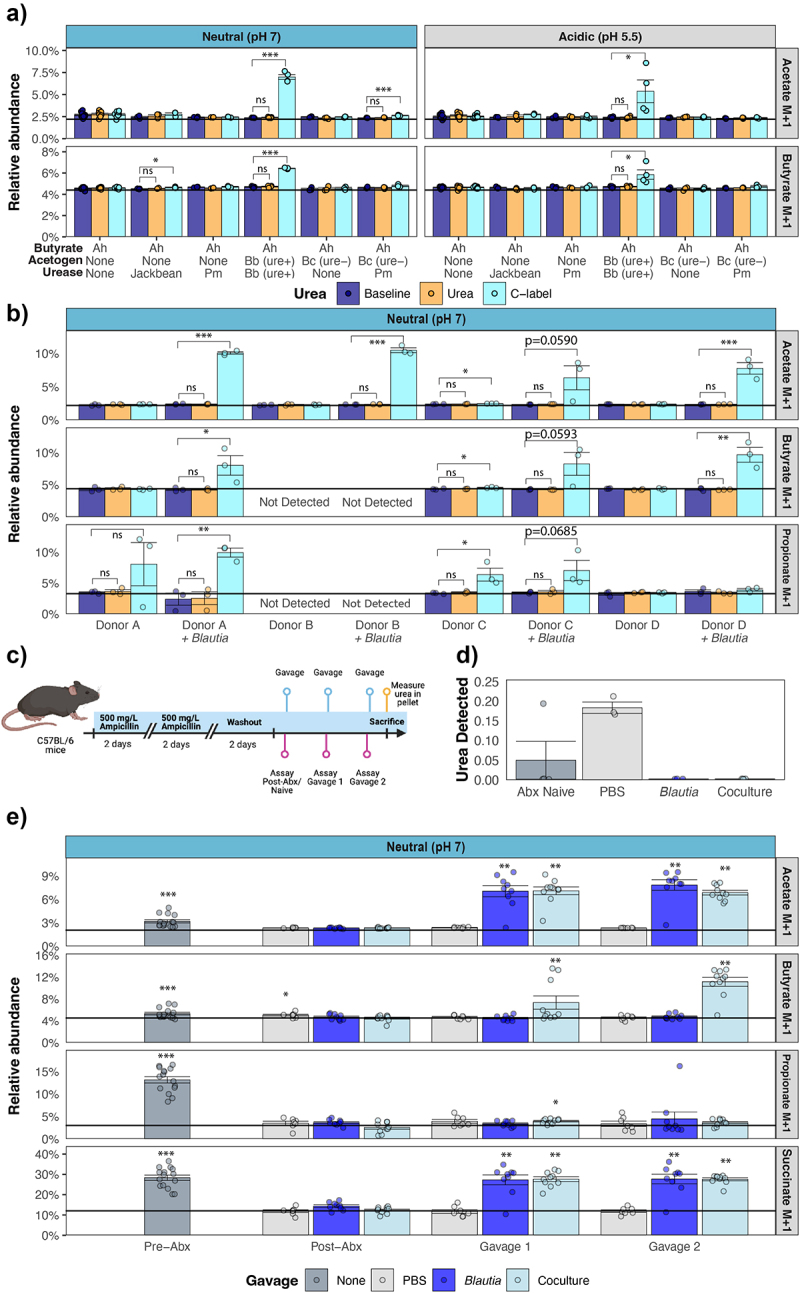
Urea-derived acetogenesis allows for cross-feeding to other SCFA in communities. a) Relative abundance of heavy (+1) isotopes of acetate and butyrate from ^13^C-urea supplemented cocultures of indicated strains with *Anaerostipes hadrus*. b) Relative abundance of heavy (+1) isotopes of acetate, butyrate and propionate from 6-hour ex vivo cultures of healthy human stool with or without the presence of urease-encoding *Blautia*. Solid horizontal lines indicate the baseline abundance of the given isotope. c) Schematic of mouse experiment. Created in BioRender. d) Normalized peak area of urea from mouse pellets harvested at sacrifice. e) Relative abundance of heavy (+1) isotopes of acetate, butyrate, propionate and succinate from ex vivo assays of mouse pellets. Solid horizontal lines indicate the natural or baseline abundance of the given isotope. (*p < 0.05, **p < 0.01, ***p < 0.001).

Next, we asked if *Blautia’s* ability to salvage urea carbon in acetate results in distribution of urea carbon into the broader SCFA pool, including butyrate, through cross-feeding interactions. First, we tested if urea-derived acetate was used in cross-feeding interactions with an isolate of *Anaerostipes hadrus*, another *Lachnospiraceae* species that uses acetate to form butyrate.^20^ In the absence of acetogenic *Blautia*, urease- and acetogenesis-negative *Anaerostipes* monocultures did not incorporate urea carbon into butyrate with ^13^C-labeled urea supplementation with or without exogenous urease supplementation (Figure 7(a)). In contrast, cocultures of *Anaerostipes* and urease-encoding *Blautia* isolates in the presence of ^13^C-labeled urea resulted in significant ^13^C-labeling of butyrate (Figure 7(a)). Co-culture with the urease negative but acetogenic *Blautia* isolate did not result in ^13^C-labeling of butyrate (Figure 7(a)). In line with our results in monocultures, incorporation of urea carbon into butyrate was less efficient if urease activity was encoded by a third microbe, *Proteus* MH42F (Figure 7(a)).

To determine if urea-derived acetogenesis and subsequent cross-feeding occurs in complex communities, we first used fecal samples from healthy human donors. Metagenomic sequencing of these communities revealed variable community composition and the relative abundance of *Blautia* varied extensively (Fig S4A). Furthermore, urease and acetogenesis gene abundances varied between samples (Fig S4BC). While acetogenesis reads mapped to *Blautia*, urease reads mapped to *Streptococcus spp*., suggesting that these donors were not colonized with ureolytic *Blautia*. This allowed us to compare the salvage of urea carbon in SCFAs with or without the introduction of urease-encoding *Blautia* in an *ex vivo* assay. In line with varying community composition, we observed donor-specific SCFA production profiles, and introduction of ureolytic *Blautia* increased acetate production (Fig S4D). In the absence of urease-encoding *Blautia*, propionate-producing communities (Donors A, C, D) incorporated urea carbon in propionate – consistent with CO_2_ incorporation by phosphoenolpyruvate (PEP) carboxylase activity (Fig S5). Ureolytic *Blautia* in the community enabled urea carbon salvage into acetate (Figure 7(b)). Furthermore, for all communities that produced butyrate (Donors A, C, D), urea carbon was detected in butyrate, indicative of cross-feeding between ureolytic *Blautia* and butyrate producers (Figure 7(b)). Overall, this demonstrates that the ureolytic *Blautia* enables urea-dependent acetogenesis and cross-feeding in complex communities. Furthermore, in communities with ureolytic *Blautia*, urea carbon is salvaged in all three major SCFAs (Fig S5).

Next, we asked whether the microbiome’s salvage of urea carbon in SCFAs could be modulated using a mouse model (Figure 7(c)). Endogenous urea levels were low in colon contents from most antibiotic naïve mice which is consistent with microbial urease activity (Figure 7(d)). Therefore, we assessed the capture of ^13^C urea carbon in SCFA in fecal pellets from antibiotic naïve mice. As expected, antibiotic naïve fecal communities have the capacity to produce acetate, propionate and butyrate (Fig S4E). Similar to human donor samples, urea carbon was incorporated into propionate (Figure 7(e)). Interestingly, urea carbon was also incorporated in acetate for a subset of mice, consistent with endogenous urease and acetogen activity (Figure 7(e)). Following antibiotic treatment, endogenous urea levels were elevated in colon contents, indicating a loss of urease activity (Figure 7(d)). Antibiotic treatment reduced SCFA production and eliminated the salvage of urea carbon into SCFA pools (Figure 7(e), S4F). Colonization with orally administered ureolytic *Blautia*, or ureolytic *Blautia* with *Anaerostipes* resulted in a near-complete reduction of endogenous urea in colon contents (Figure 7(d)). This shows that, *in* *vivo*, urease in acetogenic *Blautia* is expressed and active. Furthermore, colonization with *Blautia* or *Blautia* and *Anaerostipes* led to a recovery in the capacity to produce acetate or acetate + butyrate, respectively (Fig S4F). Importantly, in these samples, urea carbon was salvaged in acetate (*Blautia* alone) or acetate and butyrate (*Blautia* + *Anaerostipes*) (Figure 7(e)). Altogether, this demonstrates that the capacity to capture urea carbon in SCFA pools exists at baseline, can be lost following antibiotic treatment, and can be manipulated through the introduction of different *Lachnospiraceae* isolates.

## Discussion

SCFA production and pH modulation are important functions of the gut microbiota that influence community composition and colonization resistance.^5,6^ Here, we found that urease genes are associated with greater acidification in *Lachnospiraceae* (Figure 1(a,b)). This led us to examine urease in *Blautia* spp. which are acetogenic members of *Lachnospiraceae*. Interestingly, in terms of gene cluster arrangement and sequence similarity, *Blautia* urease is similar to the well-characterized urease in *Helicobacter* (Figure 2(a,e)). Like *Helicobacter*, *Blautia* encode a urea transport channel – ureI. Interestingly, *Blautia* do not encode the same extracellular ureI loop that mediates pH gating in *Helicobacter* but do have a distinct extracellular loop region that remains to be characterized and is not observed in the non-pH gated, *Streptococcus* channel (Figure 2(b,c)).

The similarity to *Helicobacter* urease and association with acidification led to the hypothesis that *Blautia* use urease to survive pH/SCFA stress. In agreement, urease-encoding *Blautia* showed increased viability with urea supplementation, in a urease-dependent manner (Figure 3(a)). Interestingly, the concentration of urea required to increase viability was dependent on the initial pH of the culture. In neutral starting conditions, viability increased with urea concentrations as low as 2.5 mM, whereas 10 mM urea was necessary to increase viability in acidic conditions. Studies examining urease function, including in other gut residents, have used a range of urea concentrations including up to 200 mM for studies of *Bifidobacterium longum* subsp. *infantis* metabolism of urea supplied in breast milk.^17^ In adulthood, up to 20% of host urea is released into the gut, and, although measuring steady-state urea concentrations is challenging because of microbial metabolism, measurements indicate enteric urea concentrations that agree with the range of urea concentrations applied here (1–10 mM).^45^ While we found that urea increased our *Blautia* strains’ viability, it will also be interesting to determine if *Blautia* urease impacts the replication and survival of other community members under acid stress and whether effects are community-wide or require close interaction between *Blautia* and other commensals. Furthermore, not all *Blautia* isolates encode urease or respond to urea supplementation. This suggests the existence of additional, yet uncharacterized, mechanisms of pH stress responses in *Blautia* and *Lachnospiraceae.*

We found that urease activity in *Blautia* increases acetate production (Figure 5). Unexpectedly, this can occur independent of changes in viability, consistent with a shift in metabolism. Several studies have investigated the effects of urease on fermentation in gut microbes. In bifidobacteria, urea supplementation alters growth and nitrogen utilization pathways with downstream, indirect effects on fermentation metabolite production.^17^ Similarly, urease-encoding *Streptococcus thermophilus* isolates show increased production of lactic acid when supplemented with urea, results that are recapitulated with ammonia supplementation,^18^ indicating that it is the regulatory role of urea-derived ammonia that is affecting carbohydrate metabolism. In contrast, we found that acetogenic *Blautia* isolates directly incorporate the urea carbon into acetate and succinate (Figure 6(b)). The increase in abundance of +2 acetate when both ^13^C-urea and ^13^C-formate are present indicates that acetogenesis is used to salvage urea carbon. Further, we found that this combination of urease and acetogenesis capabilities is rare across gut microbe species (Figure 6(f)). Indeed, *Blautia* spp. are the predominant taxa with this capability. We then examined if these functions were active *in* *vivo*. Analysis of metatranscriptomic data from the HMP dataset demonstrated i) expression of urease and acetogenesis genes is widespread in human samples, and ii) that co-expression is almost entirely unique to *Lachnospiraceae* and *Blautia* (Figure 6(g)). Interestingly, isolates of other *Lachnospiraceae* genera encode urease (Figure 1(d)) and show co-occurrence of urease and acetogenesis gene transcription in human microbiomes (Figure 6(g)). It will be interesting to determine if urease plays a similar role in these related *Lachnospiraceae* as in *Blautia*. Overall, this indicates that the gene expression necessary to salvage urea-carbon in acetate occurs naturally in the human gut microbiota. In agreement, colonization of mice with ureolytic *Blautia* strongly reduced urea availability in the colon (Figure 7(d)). Altogether, this demonstrates that the functions required for urea-derived acetogenesis are active *in* *vivo*.

Next, we asked if the salvage of urea-carbon in acetate occurs in complex communities. In agreement, an earlier study using IP administration of ^13^C,^15^N-urea to hibernating squirrels, demonstrated that urea is transported to the gut where it is processed by microbial urease.^46^ Importantly, in this model, urea carbon is detected in acetate, but the mechanisms of urea-carbon incorporation into acetate were not investigated. Here, we found that, like squirrels, a subset of antibiotic naïve mice showed incorporation of urea carbon into acetate (Figure 7(e)). Furthermore, we demonstrate that the introduction of ureolytic *Blautia* in either human communities *ex vivo* or through colonization of antibiotic treated mice leads to significant salvage of urea carbon in acetate (Figure 7(c–e)). While the incorporation of urea carbon in acetate is significant, it is important to note that acetate is the most abundant SCFA, is produced by most gut resident bacteria, and 90–95% of SCFA are absorbed along the length of the colon.^47^ Indeed, compared to butyrate or propionate, *in silico* predications of acetate production levels by communities are challenging.^48^ As a result, assessing the contributions of individual taxa to the overall pool of this abundant metabolite is also difficult. However, as acetogens, members of *Blautia* can produce proportionally higher levels of acetate than other taxa, and our data indicate that a substantial fraction of this *Blautia*-produced acetate can be derived from urea.

Acetate is used in cross-feeding interactions. In line with this, when isolates of ureolytic *Blautia* and *Anaerostipes hadrus* are co-cultured, urea-derived acetate is incorporated into butyrate (Figure 7(a)). This mechanistically shows that cross-feeding interactions enable recycling of urea-derived carbon into broader metabolite pools. We then asked if this cross-feeding occurs in complex communities. In agreement, the presence of ureolytic *Blautia* enabled the incorporation of urea carbon into butyrate by both human and mouse microbiotas. Given acetate ubiquity in the gut, involvement in other metabolic processes, and cross-feeding interactions, it is likely that urea-carbon is present in other metabolite pools beyond butyrate studied here. Therefore, future work identifying the broader dissemination of urea carbon through other pathways after acetogenesis will be informative. Similarly, the fate of urea-nitrogen in *Blautia* remains to be investigated.

We observed that, in human stool communities without ureolytic *Blautia*, urea carbon can be incorporated into succinate and propionate. This is consistent with activity of PEP carboxylase, which is widespread and was detected in the human fecal samples used here (*data not shown*). Indeed, *Blautia* themselves incorporate urea carbon into succinate and encode PEP carboxylase (Figure 5 and *data not shown*). Interestingly, the presence of ureolytic *Blautia* leads to urea carbon salvage in the three most abundant SCFA; acetate, propionate, and butyrate in human stool samples. Our data also reveal that non-ureolytic but acetogenic *Blautia* can incorporate urea carbon released in the extracellular environment or by other urease-encoding microbes into acetate, albeit at lower efficiencies than in ureolytic *Blautia*. Therefore, it will be informative to investigate additional human microbiota compositions that vary in the taxa that encode urease and acetogenesis, and produce propionate, butyrate and succinate.

Our finding that *Blautia* spp. can encode a functional urease that alters pH regulation and SCFA has several implications for host health. First, in contrast to ureolytic *Blautia*, ureolytic but non-acetogenic *K. pneumoniae* MH258 and *P. mirabilis* MH42F did not incorporate urea carbon into SCFA (Figure 6(b)). Furthermore, *K. pneumoniae* and *P. mirabilis* significantly increased the pH with urea supplementation, while ureolytic *Blautia* continued to acidify media in the presence of urea (Figures 3, 4). Therefore, there is a difference in the outcomes of urease activity in different taxa. By extension, in IBD, when urease expression shifts from commensals, including *Blautia*, to Pseudomonadota,^15^ this will result in a change in the outcomes of urease activity. In addition, ureolytic *Blautia’s* strong depletion of endogenous urea suggests that *Blautia* may also function to limit urea availability for ureolytic Pseudomonadota that expand in the setting of IBD. Similarly, colonization with non-ureolytic *Blautia* might increase the relative availability of urea to pathogens. Indeed, the ability of *Blautia* to use urea and produce increased acetate without raising the pH under acidic conditions could contribute to the high SCFA/low pH environment that supports colonization resistance.^4,5^

Our results indicate that urea carbon salvage through acetogenesis occurs in the context of complex communities, however, this pathway could play an even larger role in the context of a limited diversity setting where individual microbiome members make up a larger portion of the community. Therefore, these mechanistic insights into *Lachnospiraceae* metabolism are informative for the rational development of defined consortia as live biotherapeutic products (LBPs), which are often intended or required to function post microbiota disruption. *Lachnospiraceae* are common constituents of in development of LBPs. For example, the VE303 consortia for treating recurrent *Clostridioides difficile* infections^49^ contains eight isolates, five of which are *Lachnospiraceae*. We show here that there is heterogeneity between *Blautia* isolates and that urease is likely an important parameter to consider in LBP design. The inclusion or exclusion of acetogenic *Blautia* that are also ureolytic in future LBPs will impact urea availability, *Blautia* survival in different pH conditions, and recycling of urea carbon in SCFA pools produced by the LBP.

## Supplementary Material

Supplemental Material

## Data Availability

All data required to assess the conclusions in Figures 1–7 and S1-S4 are present within the paper and supplemental figures. Sequencing data presented in Figure S4 are accessible as Bioproject #: PRJNA1217924
