# Supplementary material for: Urease in acetogenic Lachnospiraceae drives urea carbon salvage in SCFA pools
Source: Gut Microbes. 2025 Apr 15;17(1):2492376. doi: 10.1080/19490976.2025.2492376 (PMC12001548; doi:10.1080/19490976.2025.2492376)
Supplement: Supplemental Material [file KGMI_A_2492376_SM0939.zip › Firth_et_al_2025Supp.docx]

**
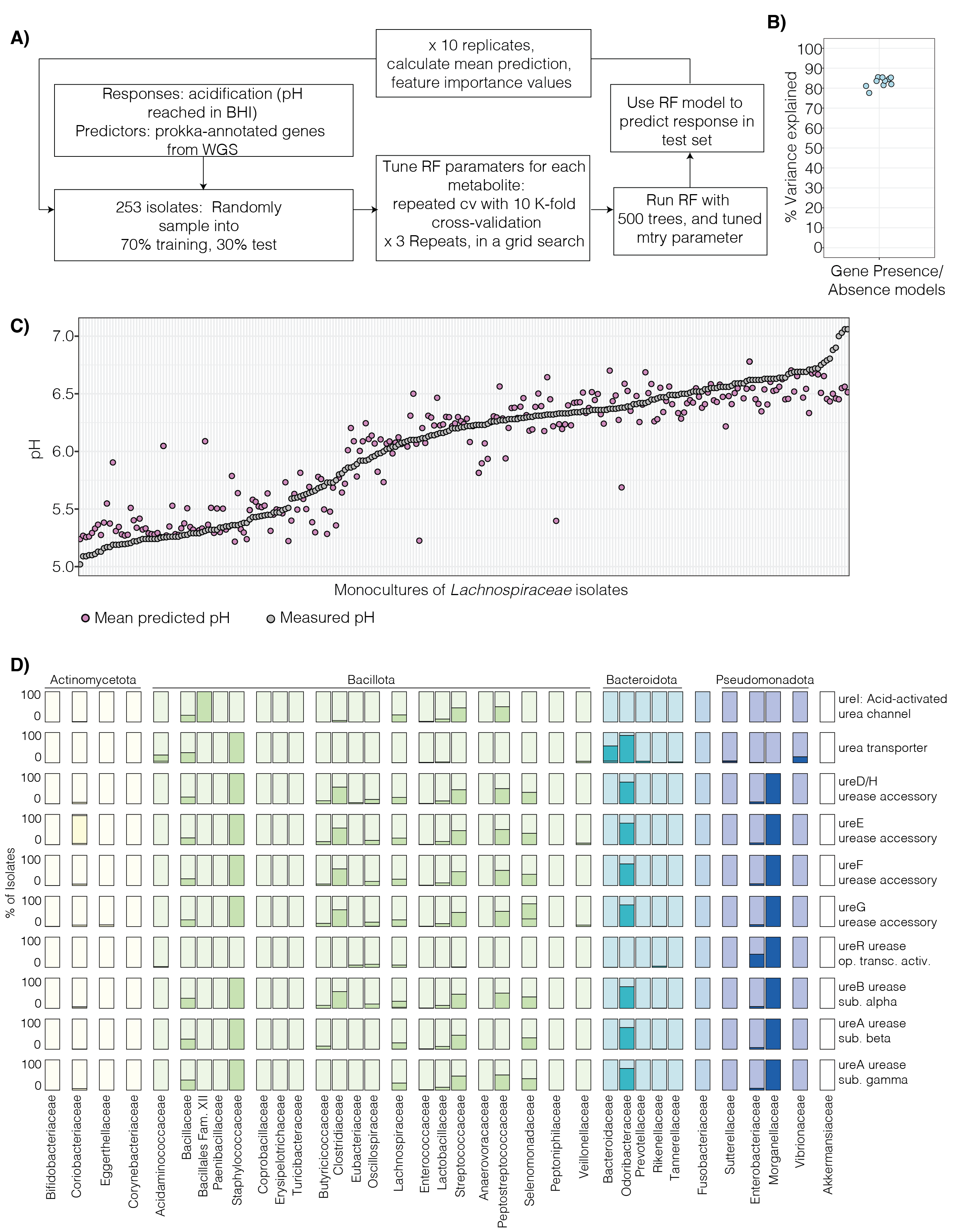
**

**Figure S1**

**
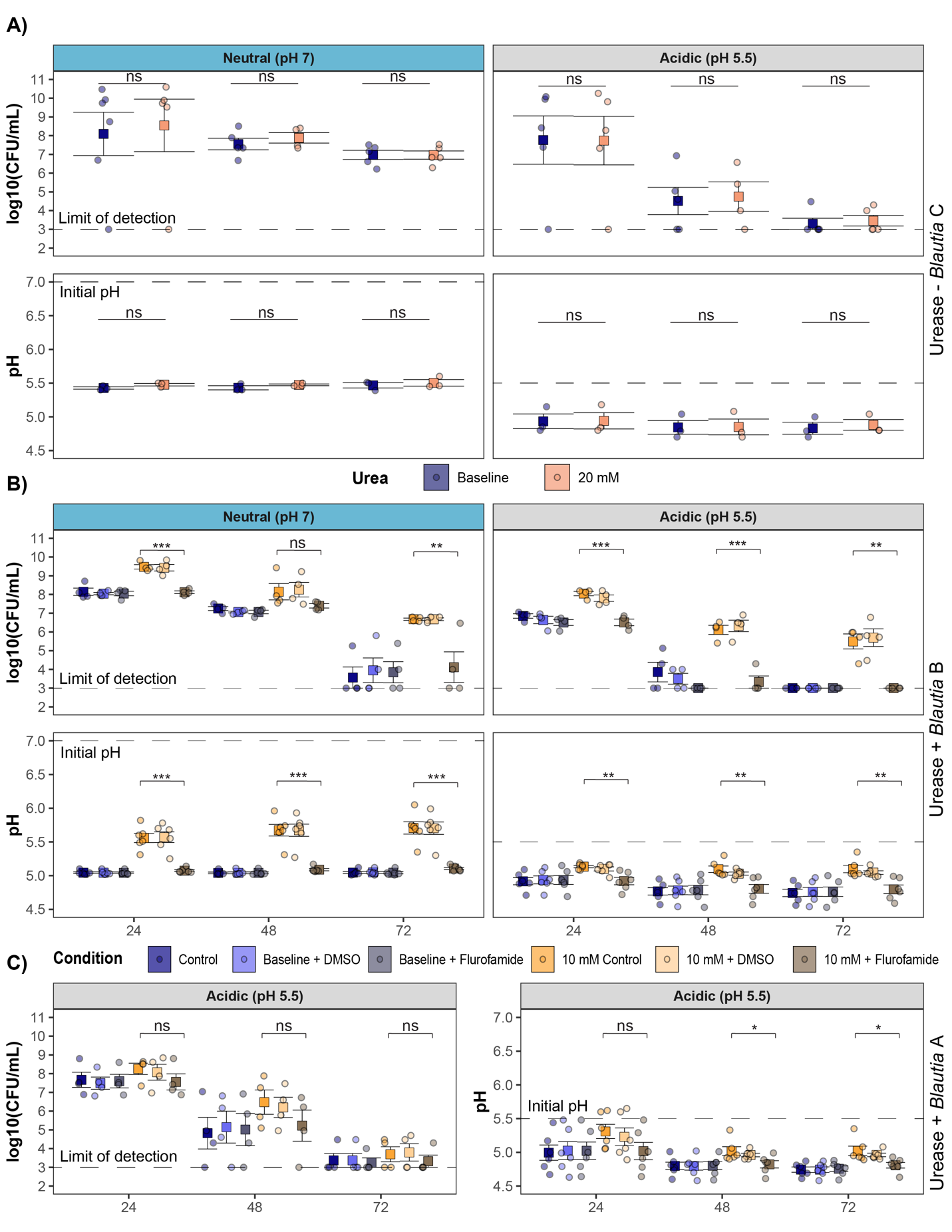
**

**Figure S2**

**
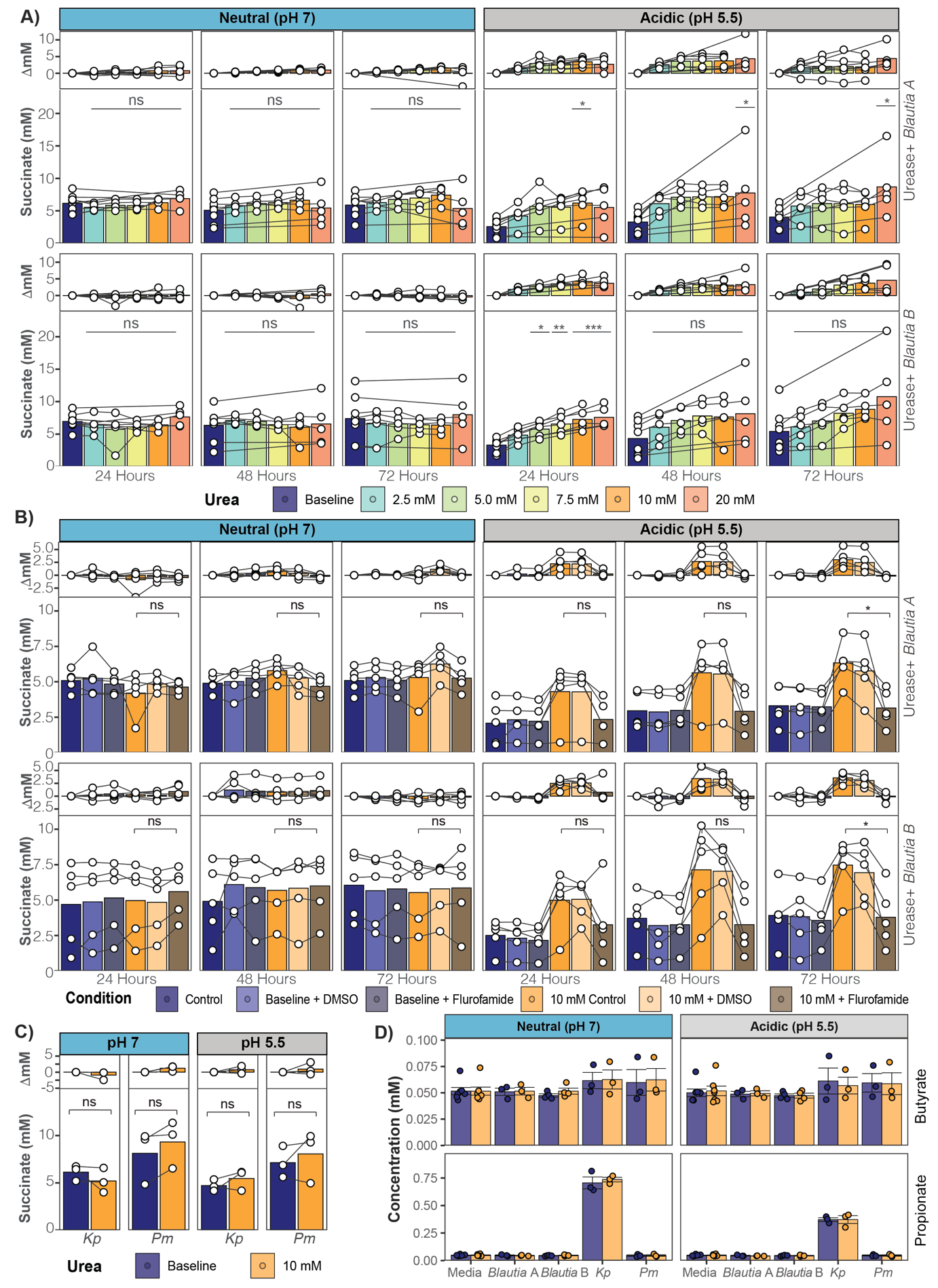
**

**Figure S3**

**
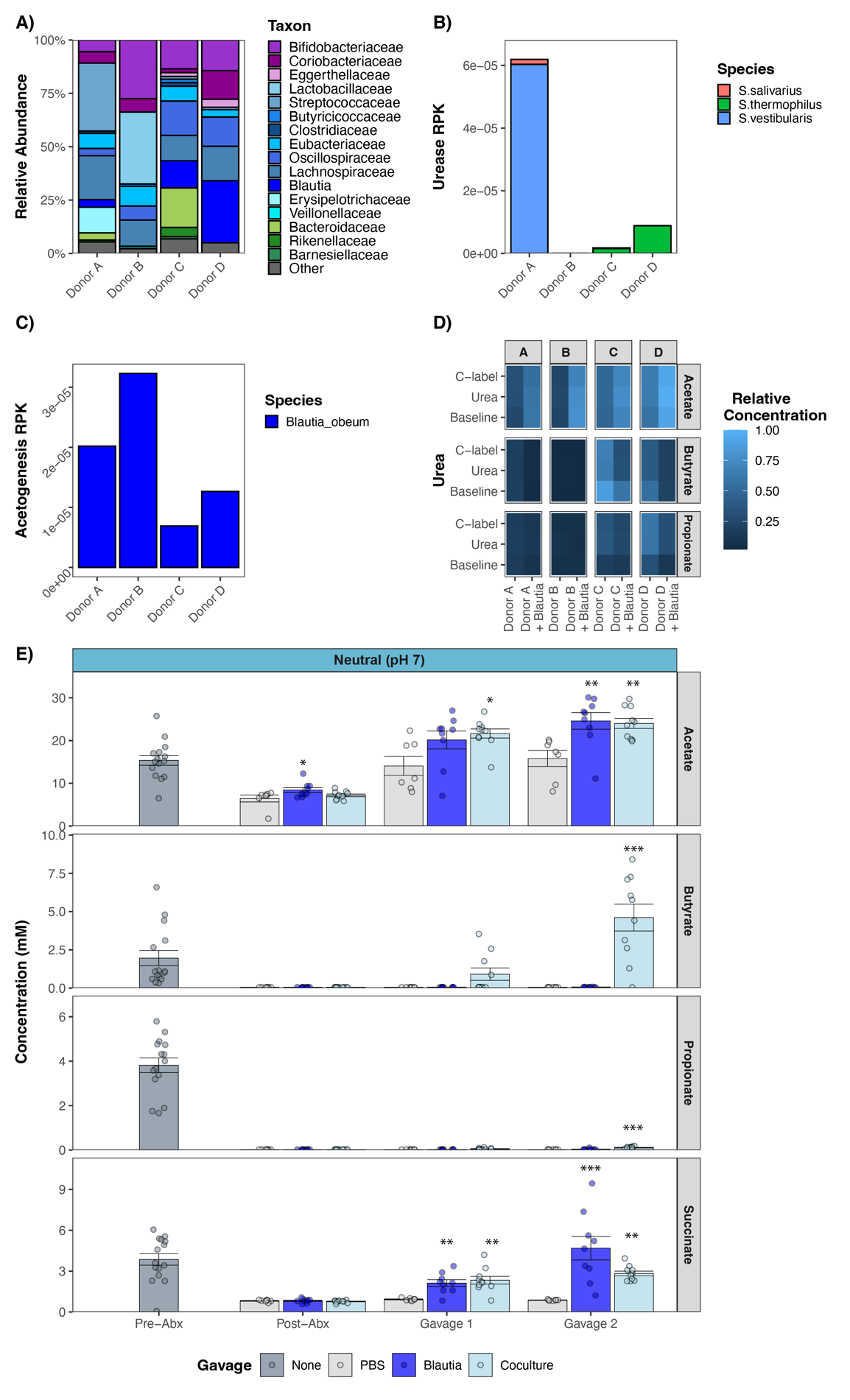
**

**Figure S4**

**
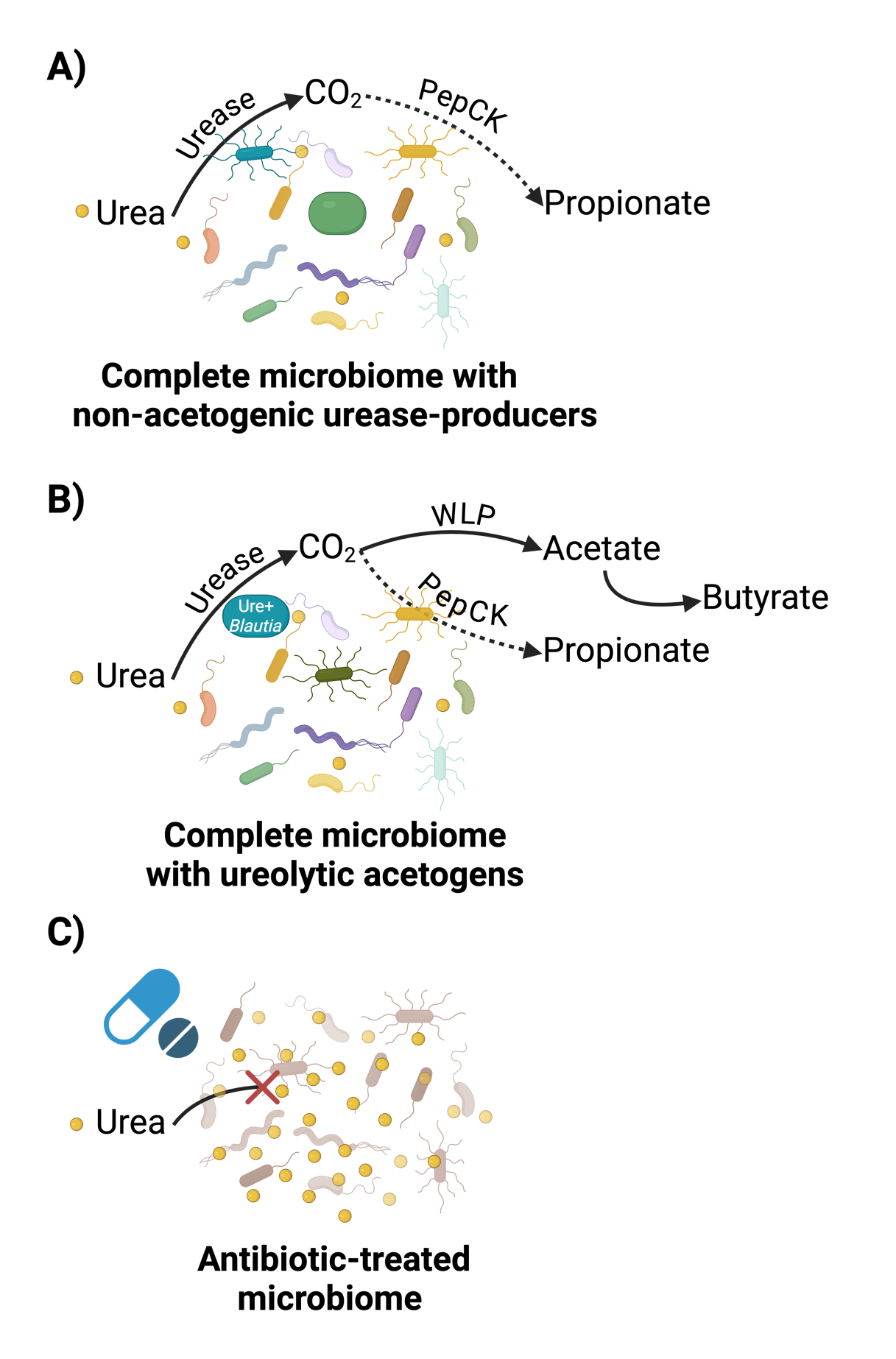
**

**Figure S5**

**Supplementary Figures**

**Figure S1. Random forest model and gene presence. A)** Schematic showing random forest (RF) procedure. **B)** Percent variance of the RF models explained by presence/absence models for 10 replicates **C)** Mean of model predicted pH values compared to measured culture pH values. **D)** Percentage of isolates in different families with urease genes across 4901 gut isolates as described in Methods.

**Figure S2. Viability and acidification of *Blautia* isolates in response to urea supplementation and urease inhibitor.** **A)** Viability and acidification of a urease-negative *Blautia* isolate in neutral or acidic starting pH conditions with 20 mM urea supplementation. **B)** Viability and acidification of urease-positive *Blautia* B with urea supplementation and urease inhibitor (flurofamide) or vehicle (DMSO). **C)** Viability and acidification of urease-positive *Blautia* A in acidic conditions with urea supplementation and urease inhibitor (flurofamide) or vehicle (DMSO). Data points represent individual samples from independent replicates. (* p<0.05, ** p<0.01, *** p<0.001)

**Figure S3. Production of succinate and other SCFA by urease-encoding isolates. A)** Production of succinate by urease-positive *Blautia* in neutral or acidic starting pH conditions with various urea supplementations. **B)** Succinate production of urease positive *Blautia* with urease inhibitor. **C)** Succinate production by urease positive *K. pneumoniae* (Kp) and *P. mirabilis* (.m). The change in concentration (upper panels) was calculated within each experimental replicate, between the treatment conditions. D) Production of butyrate and propionate from urease-positive *Blautia* and ureolytic pathogens supplemented with urea. Data points represent individual samples from independent replicates, lines connect samples within replicates. (* p<0.05, ** p<0.01, *** p<0.001)

**Figure S4. Metagenomic data of human fecal samples and SCFA production of human and mouse ex vivo cultures.** A) Taxonomic composition, B) urease abundance and C) acetogenesis abundance generated from shotgun sequencing of Donor fecal samples. D) Donor SCFA profiles from human ex vivo cultures. Relative concentration was calculated as proportion of max concentration for that SCFA. E) Production of SCFA from mice colonized with the indicated *Lachnospiraceae* ex vivo. (* p<0.05, ** p<0.01, *** p<0.001)

**Figure S5. Proposed mechanism of urea-carbon salvage driven by ureolytic acetogens.** A) In a complete microbiome with non-acetogenic urease producers, host-derived urea (yellow circles) is cleaved into CO_2_ by microbial urease, followed by incorporation into propionate pools, possibly via PEP carboxylase activity (PepCK). B) In a complete microbiome with ureolytic acetogens such as *Blautia* (blue), host-derived urea is cleaved to CO_2_ and broadly disseminated into SCFA pools by either acetogenesis (WLP) and butyrate cross-feeding, or PEP carboxylase activity. C) In an antibiotic-depleted microbiome, microbial urease activity is decreased, and host-derived urea accumulates in the gut environment. Created in BioRender.
